# Hospitalized children with COVID-19 infection during large outbreak of SARS-CoV-2 Omicron strain: a retrospective study in Chaozhou, Guangdong, China

**DOI:** 10.1080/07853890.2024.2389301

**Published:** 2024-08-10

**Authors:** Fen Lin, Man-Tong Chen, Lin Zhang, He Xie, Zhe Yang, Bin Huang, Jian-Peng Wu, Wei-Hao Lin, Li-Ye Yang

**Affiliations:** a Precision Medical Lab Center, Chaozhou Central Hospital Affiliated to Southern Medical University, Chaozhou, PR China; b Department of Pediatrics, Chaozhou Central Hospital Affiliated to Southern Medical University, Chaozhou, PR China; c Precision Medical Lab Center, People’s Hospital of Yangjiang, Yangjiang, PR China

**Keywords:** Omicron wave, paediatric patients, clinical characteristics, febrile convulsions, multisystem inflammatory syndrome in children (MIS-C)

## Abstract

**Objective:**

We aimed to investigate the clinical findings of hospitalized paediatric COVID-19 patients by the end of 2022.

**Method:**

All confirmed children with COVID-19 infection admitted into Chaozhou Central Hospital during the COVID-19 outbreak from 19 December 2022 to 1 February 2023 were included. Detailed clinical data of those children were evaluated retrospectively.

**Results:**

A total of 286 children, ranging in age from 1 month to 13 years old, were diagnosed with SARS-CoV-2 infection. Among these cases, 138 (48.3%) were categorized as mild, 126 (44.0%) as moderate and 22 (7.7%) as severe/critical. Symptoms varied among the children and included fever, upper respiratory tract symptoms, convulsions, sore throat, poor appetite, dyspnoea and gastrointestinal symptoms. Notably, febrile convulsions were observed in 96 (33.6%) patients, while acute laryngitis was documented in 50 (17.5%) cases. Among the severe/critical patients, eight developed multisystem inflammatory syndrome in children (MIS-C), and tragically, one patient’s condition worsened and resulted in death. Furthermore, MRI scans revealed abnormal brain signals in six severe/critical patients. The severe/critical group also exhibited more pronounced laboratory abnormalities, including decreased haemoglobin and elevated ALT, AST, LDH and CK levels.

**Conclusions:**

Febrile convulsions and acute laryngitis are frequently observed in children diagnosed with SARS-CoV-2 Omicron infection. Moreover, MIS-C and abnormal neuroimaging appear to be relatively common phenomena in severe/critical cases.

## 1. Introduction

The emergence of the SARS-CoV-2 Omicron variant (B.1.1.529) in South Africa on 9 November 2021, initiated a rapid global spread of the virus. The Omicron genome has formed a distinct class, characterized by over 30 mutations in the spike protein. This accumulation of mutations has enhanced the virus’s ability to bind to human cells, resulting in increased infectivity and immune evasion. Consequently, the prevention and control of coronavirus disease have become even more challenging [1,2].

China has been implementing ‘zero COVID’ strategies since August 2021 to combat SARS-CoV-2 infection, which refers to an integration of comprehensive prevention and control measures to quickly extinguish the epidemic when local cases occur. The Chinese government has been adjusting and optimizing epidemic prevention and control measures based on the evolving situation. Towards the end of 2022, the outbreak of SARS-CoV-2 Omicron variant has been spreading relentlessly across China. Due to the increased transmissibility of the SARS-CoV-2 Omicron variant, a significant surge in hospitalizations has placed a strain on healthcare systems. During this period, there has also been a notable increase in the total number of hospitalizations of children infected by the Omicron variant. While most paediatric cases exhibit milder symptoms, clinical manifestations can vary depending on factors such as age and underlying health conditions. Chaozhou, located in eastern Guangdong province, has never experienced a COVID-19 pandemic, and the whole population was naive to this virus. Furthermore, children under the age of 3 years did not receive the COVID-19 vaccine in China. These factors may contribute to the distinct presentation of COVID-19 in children compared to other countries.

This retrospective study provides an overview of 286 children with SARS-CoV-2 Omicron infection who received treatment at the paediatric department of Chaozhou in eastern Guangdong Province, China. Among these cases, eight children developed multisystem inflammatory syndrome (MIS). This paper has been made available previously as a preprint. Hyperlink with https://doi.org/10.21203/rs.3.rs-3170038/v1

## 2. Materials and methods

### 2.1. Study design and patients

The study included a total of 286 cases of children ranging in age from 1 month to 13 years. These cases were admitted to the hospital during the COVID-19 outbreak between 19 December 2022 and 1 February 2023. Laboratory confirmation of SARS-CoV-2 infection was determined by a positive result from RT-PCR or rapid antigen testing (RAT) on throat swab samples. The study followed the National Health Commission of China’s updated diagnosis and treatment guidance for novel coronavirus infection (10th edition, trial), which provides a standardized approach to diagnosing and classifying the severity of SARS-CoV-2 infection (mild, moderate and severe/critical) for appropriate management and treatment [3]. The criteria were as follows: (1) mild: the main symptoms are upper respiratory tract infections, such as sore throat, cough and fever, etc.; (2) moderate: continuous high fever >3 days or (and) coughing, shortness of breath, etc., respiratory rate (RR) <30 times/min; the oxygen saturation was greater than 93% when inhaling air in a resting state; imaging finding showed the characteristic manifestation of COVID-19; (3) severe: defined as those meeting at least one of the major criteria, which include sustained high fever >3 days; symptoms of shortness of breath excluding the effects of fever and crying; the oxygen saturation is ≤93% when inhaling air in a resting state; appearance of three depression sign, wheezing; appearance of consciousness disorders or convulsions; refusal to eat or difficulty feeding, with signs of dehydration and (4) critical: meeting at least one of the major criteria, which include patients requiring mechanical ventilation for respiratory failure, patients requiring vasoactive medications for septic shock, or patients experiencing other organ failure that necessitates monitoring and treatment in the intensive care unit (ICU). Moreover, patient files were reviewed to identify cases of multisystem inflammatory syndrome in children (MIS-C) based on the diagnostic criteria of WHO [4].

### 2.2. Data collection

All case report forms were coded before analysis to ensure anonymity, complying with the Declaration of Helsinki and the Habeas Data Law on personal data protection. The study was approved by the institutional ethics board of Chaozhou Central Hospital (No. 2023010).

Information including demographic data, medical history, underlying comorbidities, symptoms, signs, laboratory findings, radiology examinations and treatment measures (such as antiviral therapy, immunotherapy and respiratory support) were collected. The date of disease onset was defined as the day when symptoms were first noticed. Clinical outcomes recorded in the study include patient cure/discharge and mortality.

### 2.3. Laboratory confirmation

SARS-CoV-2 infection was conducted at the Department of Clinical Laboratory of Chaozhou Central Hospital. RNA extraction and RT-PCR assay were performed following the manufacturer’s protocol using a fluorescence-based quantitative PCR kit. Additionally, a commercial SARS-CoV-2 antigen rapid test kit was utilized to detect the presence of the viral antigen. Since clinicians suspected that some patients may have been infected with other respiratory viruses in addition to COVID-19, or that their clinical condition was unstable despite being treated as COVID-19 cases, 113 patients were tested for six respiratory viruses using PCR assays. These respiratory viruses included influenza virus (A and B), parainfluenza virus, respiratory syncytial virus, rhinovirus and adenovirus.

### 2.4. Statistical analysis

Statistical charts or boxplots were constructed using Prism 5 (GraphPad Software, La Jolla, CA). Other statistical analyses were performed using SPSS Software V19.0 (IBM Corp., Armonk, NY). Basic demographic and clinical information were presented as median values with interquartile range (IQR) for continuous variables and percentage for categorical variables. The laboratory data were presented as *n* (%) or mean (SE). The total number of patients with available data for each item was indicated in the table. Differences of continuous variables between the mild, moderate and severe/critical groups were analysed using independent group *t*-tests when the data followed a normal distribution; otherwise, the Mann–Whitney test was employed. Differences of categorical variables between the groups were compared using the *χ*^2^ test or Fisher’s exact test. All statistical tests were two-sided, and a *p* value of less than .05 was considered statistically significant.

## 3. Results

### 3.1. Clinical features

**Figure 1.**
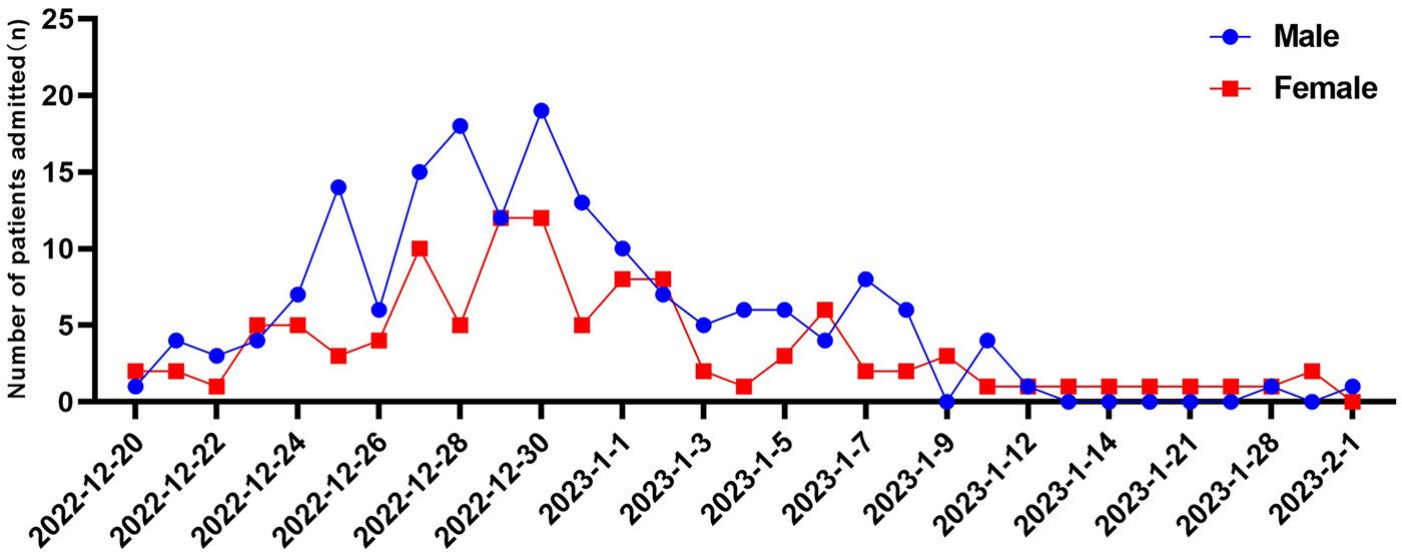
The number of daily admitted cases in the paediatric department.

From 20 December 2022 to 1 February 2023, 286 children with COVID-19 were admitted in our hospital (Figure 1). The length of hospital stay for severe/critically ill patients was 7.0 (4.5–9.3) days, which showed significant statistical differences compared to mild and moderate patients (*p* < .01) (Table 1). Out of the 286 cases, 256 (89.5%) presented with fever, with 174 (60.8%) patients having a temperature of ≥39 °C, and 35 (12.2%) experiencing fever for ≥5 days. Upper respiratory symptoms, including cough, sore throat, runny nose and nasal congestion, were observed in most children. Additionally, some children experienced gastrointestinal symptoms such as vomiting, nausea and diarrhoea. Fifty (17.5%) children had acute laryngitis accompanied by cough and antiadoncus. Moreover, 96 (33.6%) patients experienced febrile convulsions, with 13 (59%) of these cases classified as severe/critical. Additionally, 13 (4.5%) patients had comorbidities, and four of these cases developed into severe/critical illness (Table 1).

**Table 1.** Demographic and clinical characteristics of 286 children with SARS-CoV-2 Omicron infection.

Characteristics	All patients	Mild	Moderate	Severe/critical	*P*1	*P*2	*P*3
Cases	286 (100)	138 (48.3)	126 (44.0)	22 (7.7)			
Gender							
Male	175 (61.2)	85 (61.6)	78 (61.9)	12 (54.5)	.959	.530	.514
Female	111 (38.8)	53 (38.4)	48 (38.1)	10 (45.5)			
Age, years at admission (median, IQR)	1.6 (0.6–2.9)	1.7 (0. 7–3.0)	1.3 (0.5–2.4)	2.6 (0.3–7.3)	.039	.188	.036
<3	218 (76.2)	102 (73.9)	104 (82.5)	12 (54.5)	.091	.062	.009
≥3	68 (23.8)	36 (26.1)	22 (17.5)	10 (45.5)			
Vaccination^c^ (age ≥ 3 years)	45 (66.2)	22 (61.1)	16 (72.7)	7 (70.0)	.366	.723	>.999
Length of hospital stay, day (median, IQR)^a^	3 (3.0–5.0)	3 (2.0–4.0)	4 (3.0–5.3)	7.0 (4.5–9.3)	<.001	.001	.009
Fever or not							
Fever	256 (89.5)	130 (94.2)	111 (88.1)	15 (68.2)	.094	.001	.024
Peak temperature ≥39 °C	174 (60.8)	87 (66.9)	80 (72.1)	7 (46.7)	<.001	.495	.071
Fever ≥ 5 d	35 (12.2)	11 (8.1)	22 (19.8)	2 (13.3)	.014	.184	>.999
Upper respiratory tract presentation							
Cough	225 (78.7)	101 (73.1)	108 (85.7)	16 (72.7)	.012	>.999	.205
Sore throat	18 (6.3)	8 (5.7)	9 (7.1)	1 (4.5)	.656	>.999	>.999
Antiadoncus	140 (49.0)	65 (47.1)	62 (49.2)	13(59.0)	.717	.296	.216
Runny nose	92 (32.2)	53 (38.4)	36 (28.5)	3 (13.6)	.091	.024	.142
Nasal stuffiness	52 (18.2)	28 (20.2)	22 (17.4)	2 (9.0)	.558	.375	.531
Acute laryngitis	50 (17.5)	2 (1.4)	45 (35.7)	3 (13.6)	<.001	.019	.041
Dyspnoea	54 (18.9)	15 (10.8)	33 (26.1)	6 (27.2)	.001	.046	.901
Gastrointestinal symptoms							
Vomiting/nausea	47 (16.4)	21 (15.2)	20 (15.8)	6 (27.2)	.883	.216	.225
Diarrhoea	31 (10.8)	12 (8.6)	16 (12.6)	3 (13.6)	.291	.437	>.999
Poor appetite	185 (64.7)	86 (62.3)	80 (63.4)	19 (86.3)	.844	.027	.035
Febrile convulsions	96 (33.6)	53 (38.4)	30 (23.8)	13 (59.0)	.011	.067	.001
Abnormalities on chest CT	46/64 (71.9)	0/5 (0)	35/42 ((83.3)	11/17 (64.7)	.001	.035	.166
Scattered inflammation shadowing	45/64 (70.3)	0/5 (0)	34/42 (81.0)	11/17 (64.7)	.001	.035	.197
Lung consolidation	13/64 (20.3)	0/5 (0)	7/42 (16.7)	6/17 (35.3)	>.999	.266	.166
Abnormalities on brain CT	3/35 (8.6)	0/9 (0)	0/13 (0)	3/13 (23.1)	–	.280	.232
Abnormalities on brain MRI	6/9 (66.7)	0/0 (0)	0/1 (0)	6/8 (83.3)	–	–	.333
Comorbidities	13 (4.5)	4 (2.9)	5 (4.0)	4 (18.2)	.741	.013	.028
Type 1 diabetes	2 (0.7)	0 (0)	0 (0)	2 (9.1)			
Congenital heart disease	2 (0.7)	0 (0)	2 (1.6)	0 (0)			
Others^d^	9 (3.1)	4 (2.9)	3 (2.4)	2 (9.1)			
Co-infection							
Other respiratory viruses^e^	6/113 (5.3)	3/43 (7.0)	2/56 (3.6)	1/14 (7.1)	.650	>.999	.494
MIS-C^b^	8 (2.8)	0 (0)	0 (0)	8 (36.4)	–	<.001	<.001
Medical treatment							
Antibiotics	97 (33.9)	25 (18.1)	55 (43.7)	17 (77.3)	<.001	<.001	.004
Corticosteroids	136 (47.6)	38 (27.5)	81 (64.3)	17 (77.3)	<.001	<.001	.235
Traditional Chinese medicine	84 (29.4)	39 (28.3)	42 (33.3)	3 (13.6)	.372	.148	.064
Immunoglobulin	18 (6.3)	1 (0.7)	3 (2.4)	14 (63.6)	.351	<.001	<.001
Oxygen inhalation	54 (18.8)	5 (3.6)	28 (22.2)	21 (95.5)	<.001	<.001	<.001
Nasal prong oxygen	52 (18.2)	5 (3.6)	28 (22.2)	19 (86.4)			
Mechanical ventilation	2 (0.6)	0 (0)	0 (0)	2 (9.1)			
Outcome							
Cure/discharge	285 (99.7)	138 (100)	126 (100)	21 (95.5)	–	.137	.149
Death in hospital	1 (0.3)	0 (0)	0 (0)	1 (4.5)			

*P*1: *p* values of mild vs. moderate; *P*2: *p* values of mild vs. severe/critical; *P*3: *p* values of moderate vs. severe/critical.

Data are *n* (%) except for age and length of hospital stay, where *N* is the total number of patients with available data.

^a^ Excluding four patients who have not cured and discharged from the hospital. (–) Not enough data for statistical analyses.

^b^ MIS-C: multisystem inflammatory syndrome in children.

^c^ Children without documented receipt of any COVID-19 vaccine dose before hospitalization were considered to be unvaccinated, vaccination status was confirmed from children’s parents.

^d^ Epilepsy (three cases), hyperthyroidism (one case), autoimmune anaemia (one case), cerebral palsy (one case), gastrointestinal bleeding (one case), adrenocortical hypofunction (one case) and autoimmune encephalitis (one case).

^e^ Parainfluenza virus (two cases), respiratory syncytial virus (one case), rhinovirus (two cases) and adenovirus (one case).

### 3.2. Radiologic and laboratory findings

**Figure 2.**
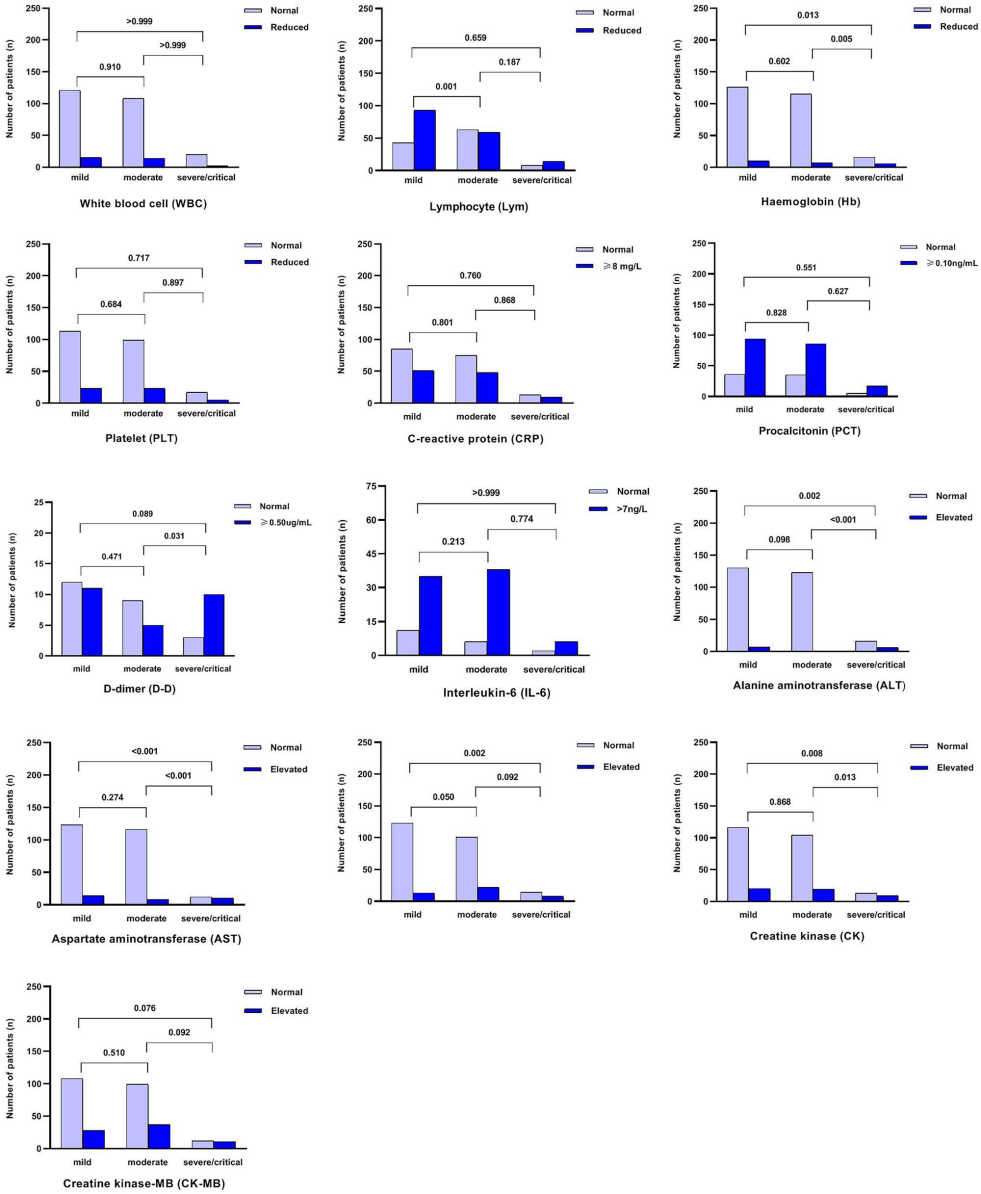
Comparison of the main laboratory indices between mild, moderate, severe and critical groups.

Radiologic findings showed that out of the 286 patients, 64 underwent chest CT examinations, with 45 of them displaying scattered inflammation shadowing (unilateral or bilateral), and 13 showing lung consolidation. Brain MRI scans revealed abnormal signals in six (66.7%) of the severe/critical patients (Table 1). Laboratory testing indicated that 81 patients had abnormal white blood cell (WBC) counts, and 166 children had a decreased total lymphocyte count upon admission. Elevated levels of C-reactive protein (CRP), procalcitonin (PCT), IL-6 and D-dimer were identified in 38.4% (108/281), 72.2% (197/273), 80.6% (79/98) and 52% (26/50) of patients, respectively. Furthermore, during hospitalization, more significant laboratory abnormalities, such as decreased haemoglobin and elevated levels of alanine transaminase (ALT), aspartate transaminase (AST) and creatine kinase (CK), were observed in the severe/critical group (mild vs. severe/critical and moderate vs. severe/critical, *p* < .05) (Figure 2).

Among 113 patients tested for the six types of respiratory viruses, two cases were positive for parainfluenza virus, one case was positive for respiratory syncytial virus, two cases were positive for rhinovirus and one case was positive for adenovirus (Table 1).

### 3.3. Treatment

During hospitalization, 54 (18.9%) children required oxygen inhalation, and antimicrobial therapy (oral or intravenous antibiotics) and corticosteroids were administered to 97 (33.9%) and 136 (47.6%) patients, respectively. Immunoglobulin therapy was given to 18 (6.3%) patients, while no patients received antiviral drugs.

Severe/critically ill patients were more likely to receive oxygen inhalation, antibiotics and immunoglobulin therapy compared to mild and moderate patients (*p* < .01). Additionally, 84 (29.4%) patients underwent Chinese traditional medicine therapy during their hospitalization (Table 1).

### 3.4. MIS in children

MIS-C is a rare hyperinflammatory disorder that occurs as a post-infectious complication of SARS-CoV-2 infection [4]. Multisystem inflammatory syndrome in children is a rare but serious condition that can occur in children and adolescents following a COVID-19 infection. It is characterized by inflammation in various body systems, including the heart, lungs, kidneys, brain, gastrointestinal organs and skin. MIS-C typically manifests several weeks after a COVID-19 infection and presents with symptoms such as persistent fever, abdominal pain, rash, red eyes, swollen hands and feet, and enlarged lymph nodes. It is believed to be an immune system reaction that occurs as a delayed response to the initial viral infection. Although rare, MIS-C requires prompt medical attention and treatment in a hospital setting [4]. Within the cohort, eight severe/critical patients developed MIS, characterized by elevated inflammatory markers such as CRP, IL-6 and PCT, along with lymphopaenia in the majority of cases. Radiographic changes in lung parenchyma were observed in four cases. All eight patients had a fever, and three required mechanical ventilation. Other clinical presentations included convulsions, hepatic injury, respiratory failure, heart failure and myocardial injury. Unfortunately, one patient deteriorated and developed respiratory failure, diffuse intravascular coagulation and septic shock, ultimately resulting in fatality. Table 2 provides an overview of the demographics, clinical symptoms, laboratory findings, imaging results, treatment regimens and outcomes of this group of eight children with MIS.

**Table 2.** The main laboratory findings of children with SARS-CoV-2 Omicron infection: mild, moderate, severe and critical groups.

Laboratory finding	All patients (*n* = 286)	Mild (*n* = 138)	Moderate (*n* = 126)	Severe/critical (*n* = 22)	*P*1	*P*2	*P*3
White blood cell (×10^9^/L)	9.64 ± 0.30	8.51 ± 0.31	10.57 ± 0.53	11.44 ± 1.33	.001	.042	.522
Reduced	31/280 (11.1%)	15/136 (11.0%)	14/122 (11.5%)	2/22 (9.1%)	.910	>.999	>.999
Elevated	50/280 (17.9%)	14/136 (10.3%)	28/122 (23.0%)	8/22 (36.4%)	.006	.003	.181
Lymphocyte (×10^9^/L)	2.64 ± 0.15	2.18 ± 0.16	3.24 ± 0.27	2.15 ± 0.41	.001	.940	.101
Reduced	166/280 (59.3%)	93/136 (68.4%)	59/122 (48.4%)	14/22 (63.6%)	.001	.659	.187
Haemoglobin (g/L)	119.07 ± 0.67	120.43 ± 0.88	117.87 ± 0.93	117.36 ± 4.12	.047	.475	.906
Reduced	23/280 (8.2%)	10/136 (7.4%)	7/122 (5.7%)	6/22 (27.3%)	.602	.013	.005
Platelet (<150 × 10^9^/L)	260.04 ± 5.30	257.09 ± 7.47	264.80 ± 8.23	251.91 ± 18.90	.488	.797	.540
Reduced	51/280 (18.2%)	23/136 (16.9%)	23/122 (18.9%)	5/22 (22.7%)	.684	.717	.897
C-reactive protein (≥8 mg/L)	108/281 (38.4%)	51/136 (37.5%)	48/123 (39.0%)	9/22 (40.9%)	.801	.760	.868
Procalcitonin (≥0.10 ng/mL)	197/273 (72.2%)	94/130 (72.3%)	86/121 (71.1%)	17/22 (77.3%)	.828	.627	.551
Interleukin-6 (>7 ng/L)	79/98 (80.6%)	35/46 (76.1%)	38/44 (86.4%)	6/8 (75.0%)	.213	>.999	.774
D-dimer (≥0.50 μg/mL)	26/50 (52%)	11/23 (47.8%)	5/14 (35.7%)	10/13 (76.9%)	.471	.089	.031
Alanine aminotransferase (U/L)	26.53 ± 0.91	26.07 ± 1.13	24.50 ± 1.10	40.86 ± 6.42	.323	.033	.020
Elevated	14/283 (4.9%)	7/137 (5.1%)	1/124 (0.8%)	6/22 (27.3%)	.098	.002	<.001
Aspartate aminotransferase (U/L)	46.39 ± 1.94	43.91 ± 1.48	42.21 ± 1.12	85.41 ± 21.09	.367	.063	.053
Elevated	32/283 (11.3%)	14/137 (10.2%)	8/124 (6.5%)	10/22 (45.5%)	.274	<.001	<.001
Lactose dehydrogenase (U/L)	327.67 ± 4.78	315.70 ± 4.95	328.07 ± 5.78	399.41 ± 39.28	.103	.046	.086
Elevated	43/281 (15.3%)	13/136 (9.6%)	22/123 (17.9%)	8/22 (36.4%)	.050	.002	.092
Creatine kinase (U/L)	177.62 ± 11.90	157.30 ± 8.56	161.99 ± 14.29	390.55 ± 110.57	.774	.048	.053
Elevated	48/281 (17.1%)	20/136 (14.7%)	19/123 (15.4%)	9/22 (40.9%)	>.868	.008	.013
Creatine kinase-MB (U/L)	22.20 ± 0.70	20.97 ± 0.67	21.63 ± 0.74	33.05 ± 6.43	.510	.076	.092
Elevated	75/281 (26.7%)	28/136 (20.6%)	37/123 (30.1%)	10/22 (45.5%)	.078	.011	.156
Creatinine (μmol/L)	38.52 ± 0.83	38.46 ± 0.98	37.07 ± 0.94	46.95 ± 6.85	.311	.233	.167
Elevated	114/281 (40.6%)	51/136 (37.5%)	56/123 (45.5%)	7/22 (31.8%)	.190	.608	.232

*P*1: *p* values of mild vs. moderate; *P*2: *p* values of mild vs. severe/critical; *P*3: *p* values of moderate vs. severe/critical.

Data are *n* (%) and mean (SE). Elevated means over the upper limit of the normal range and reduced means below the lower limit of the normal range. The normal limits: white blood cell, 0 ≤ age < 6 months: 4.3–14.2 (×10^9^/L), 6 months ≤ age < 1 years: 4.8–14.6 (×10^9^/L), 1 years ≤ age < 2 years: 5.1–14.1 (×10^9^/L), 2 years ≤ age < 6 years: 4.4–11.9 (×10^9^/L), 6 years ≤ age < 13 years: 4.3–11.3 (×10^9^/L); lymphocyte, 0 ≤ age < 6m: 2.4–9.5 (×10^9^/L), 6 months ≤ age < 1 years: 2.5–9.0 (×10^9^/L), 1 years ≤ age < 2 years: 2.4–8.7 (×10^9^/L), 2 years ≤ age < 6 years: 1.8–6.3 (×10^9^/L), 6 years ≤ age < 13 years: 1.5–4.6 (×10^9^/L); haemoglobin, 0 ≤ age < 6 months: 97–183 (g/L), 6 months ≤ age < 1 years: 97–141 (g/L), 1 years ≤ age < 2 years: 107–141 (g/L), 2 years ≤ age < 6 years: 112–149 (g/L), 6 years ≤ age < 13 years: 118–156 (g/L); platelet: 0 ≤ age < 6 months: 183–614 (g/L), 6 months ≤ age < 1 years: 190–579 (g/L), 1 years ≤ age < 2 years: 190–524 (g/L), 2 years ≤ age < 6 years: 188–472 (g/L), 6 years ≤ age < 13 years: 167–453 (g/L); C-reactive protein, <8.00 (mg/L); procalcitonin, <0.10 (ng/mL); interleukin-6, 0–7 (ng/L); D-dimer: <0.50 (μg/ml); alanine transaminase, 0 ≤ age < 1 years: 8–71 (U/L), 1 years ≤ age < 2 years: 8–42 (U/L), 2 years ≤ age < 13 years: 7–30 (U/L); aspartate transaminase, 0 ≤ age < 1 years: 21–80 (U/L), 1 years ≤ age < 2 years: 22–59 (U/L), 2 years ≤ age < 13 years: 14–44 (U/L); lactose dehydrogenase: 0 ≤ age < 1d: male 0–248 (U/L), female 0–247 (U/L), 1 d ≤ age < 1 months: male 125–735 (U/L), female 145–765 (U/L), 1 months ≤ age < 1 years: male 170–450 (U/L), female 190–420 (U/L), 1 years ≤ age < 13 years: male 155–345 (U/L), female 165–395 (U/L); creatine kinase, 0 ≤ age < 1d (U/L), 1 d ≤ age < 5 d: 26–700 (U/L), 5 d ≤ age < 6 months: 26–330 (U/L), 6 months ≤ age < 13 years: 26–229 (U/L); creatine kinase-MB, 0–24 (U/L); creatinine, 0 ≤ age < 2 years: 13–33 (μmol/L), 2 years ≤ age < 6 years: 19–44 (μmol/L), 6 years ≤ age < 13 years:27–66 (μmol/L); IVIG: intravenous immunoglobulin.

## 4. Discussion

As of 12 January 2023, there were 1.27 million coronavirus inpatients in mainland China, which had a significant strain on medical resources [5]. Earlier studies indicated that children infected with COVID-19 were less likely to be infected and experience severe symptoms compared to adults, with most cases manifesting as mild symptoms such as fever and cough [6]. However, with the increased transmissibility of the Omicron variant, the number of paediatric hospitalizations has risen. From the end of 2022 to early 2023, an epidemic of Omicron strain also occurred in Chaozhou area. Based on the hospitalization record data, most cases are concentrated between 20 December 2022 and 1 February 2023. A total of 286 children who tested positive for COVID-19 were admitted to our hospital’s paediatric department due to fever or respiratory symptoms during this period.

According to the novel coronavirus pneumonia prevention and control program (9th edition) by the China CDC, COVID-19 vaccination is recommended for individuals aged over 3 years old [7]. However, a previous study indicated that immunization with the COVID-19 vaccine provided limited protection against symptomatic disease caused by the Omicron variant [8]. Our data showed that the vaccination rate among hospitalized children aged 3 years or older was 66.2%, but there was no statistical difference in the vaccination rate among different severity groups (mild, moderate and severe/critical). The limited effectiveness of the vaccine may be attributed to the gradual waning of protection over time.

High fever has been shown to increase neuronal excitability and lower the seizure threshold, particularly in the developing central nervous system [9]. Febrile convulsions are commonly observed in children under five years old, and the exact underlying cause is still unknown. In Asia, approximately 8–10% of children experience febrile convulsions annually [10]. A report from the global-federated research network (TriNetX) revealed that 0.5% of COVID-19 patients were diagnosed with febrile seizures [11]. During the Omicron wave, we observed an increase in hospitalized children experiencing febrile convulsions. Among the children we studied, 91 (31.8%) had febrile convulsions. Although no direct neurocognitive effects were identified in these patients, this remained an unusual and noteworthy occurrence in our paediatric department. Additionally, we identified six cases with severe or critical symptoms presenting abnormal neuroimaging results (bilateral thalamic and bilateral basal ganglia symmetric hypodense lesions, or multiple abnormal signals within the brain). However, the direct effects and precise pathophysiology remain unclear as only one patient underwent a lumbar puncture, and the cerebrospinal fluid tested negative for SARS-CoV-2 RNA.

There is growing evidence that SARS-CoV-2 infection can affect the central and peripheral nervous system, particularly in children with MIS-C [12,13]. There are currently limited reports of MIS-C associated with the SARS-CoV-2 Omicron variant in China [14,15]. In our cohort, a total of eight patients were diagnosed with this condition. Among these patients, six experienced convulsions (two of whom had a history of febrile convulsions), and two were diagnosed with clinico-radiological acute necrotizing encephalopathy (ANE). The cause of ANE is likely multifactorial, resulting from factors such as hypoxia, cytokine storm, metabolic derangement, electrolyte disturbances, medication effects and potentially other immune mechanisms. ANE associated with COVID-19 infection is characterized by a rapid onset and progression, often leading to death or severe neurological sequelae. Therefore, when children infected with the novel coronavirus present with high fever or hyperpyrexia and exhibit neurological manifestations within a short period of time, ANE should be considered as a potential concern. Additionally, we observed a higher prevalence of acute laryngitis in 50 children with COVID-19. Fortunately, their symptoms rapidly improved after the administration of ambroxol or budesonide via aerosol, consistent with the pronounced inflammation caused by the Omicron variant in the upper respiratory tract. Previous studies have demonstrated that certain blood markers exhibit variations according to the severity of COVID-19 infection. These markers include WBC count, lymphocyte count, PLT count, CRP, D-dimers, IL-6, as well as AST, ALT, LDH and PCT levels [16–18]. In our study, we observed significant differences in haemoglobin levels, ALT, AST, LDH and CK between non-severe and severe/critical patients. These findings align with previous reports that have identified these markers as potential indicators of disease severity in COVID-19 patients [16–18].

## 5. Limitations

We acknowledge several limitations of our study. First, its retrospective nature limited the clinical distribution, and the sample size was small. Second, we did not conduct genetic sequencing to confirm whether the affected genotype in patients was Omicron. This was because it was not standard practice to test for specific variants of the SARS-CoV-2 virus in all patients. However, based on previous reports, it was observed that the predominant epidemic strains in China during that period were Omicron BA.5.2 and BF.7 [19,20]. Therefore, it is reasonable to infer that these patients were affected by the variant strains. Third, asymptomatic children or cases with atypical symptoms not involving respiratory symptoms or fever were not included, potentially introducing bias towards more severe cases. It is possible that mild cases were managed at home without seeking medical attention, further reducing their representation in the study cohort. Additionally, the case cohort may not be representative of the overall severity distribution of COVID-19 in children in this area, as it was biased towards more severe illnesses. Lastly, due to the unavailability of specific vaccination dates for children, we were unable to evaluate the impact of COVID-19 vaccinations on hospitalized children diagnosed with COVID-19.

## 6. Conclusions

Our study provides valuable insights into the epidemiology, virology and clinical features of SARS-CoV-2 Omicron infection in children during COVID-19 outbreak. Our findings indicate that febrile convulsions and acute laryngitis are commonly observed in infected children, while occurrences of MIS-C and abnormal neuroimaging are not rare. These findings significantly enhance our understanding of the impact of SARS-CoV-2 Omicron on children and its implications for the larger outbreak.

## Data Availability

All data generated or analysed during this study are included in this article. Further enquiries can be directed to the corresponding author.

## References

[1] Tian DD, Sun YH, Xu HH, et al. The emergence and epidemic characteristics of the highly mutated SARS-CoV-2 Omicron variant. J Med Virol. 2022;94(6):2376–2383. doi: 10.1002/jmv.27643. 35118687 PMC9015498

[2] Kandeel M, Mohamed MEM, El-Lateef HMA, et al. Omicron variant genome evolution and phylogenetics. J Med Virol. 2022;94(4):1627–1632. doi: 10.1002/jmv.27515. 34888894 PMC9015349

[3] National Health Commission of the People’s Republic of China. Novel coronavirus infection diagnosis and treatment protocol. 10th ed., trial; 2023 [cited 2023 Jan 13]. Available from: http://www.nhc.gov.cn/xcs/zhengcwj/202301/32de5b2ff9bf4eaa88e75bdf7223a65a.shtml

[4] World Health Organization. Multisystem inflammatory syndrome in children and adolescents temporally related to COVID-19; 2023 [cited 2023 Dec 7]. Available from: https://www.who.int/news-room/commentaries/detail/multisystem-inflammatory-syndrome-in-children-and-adolescents-with-covid-19

[5] Chinese Center for Disease Control and Prevention (CDC); 2023 [cited 2023 Feb 28]. Available from: https://www.chinacdc.cn/jkzt/crb/zl/szkb_11803/jszl_13141/202301/t20230115_263381.html

[6] Ludvigsson JF. Systematic review of COVID‐19 in children shows milder cases and a better prognosis than adults. Acta Paediatr. 2020;109(6):1088–1095. doi: 10.1111/apa.15270. 32202343 PMC7228328

[7] Chinese Center for Disease Control and Prevention (CDC); 2023 [cited 2023 Feb 14]. Available from: https://www.chinacdc.cn/jkzt/crb/zl/szkb_11803/jszl_11815/202206/t20220628_259881.html

[8] Andrews N, Stowe J, Kirsebom F, et al. Covid-19 vaccine effectiveness against the Omicron (B.1.1.529) variant. N Engl J Med. 2022;386(16):1532–1546. doi: 10.1056/NEJMoa2119451. 35249272 PMC8908811

[9] Smith DK, Sadler KP, Benedum M. Febrile seizures: risks, evaluation, and prognosis. Am Fam Physician. 2019;99(7):445–450. 30932454

[10] Mohamed ZA, Tang C, Thokerunga E, et al. Serum hypomagnesemia is associated with febrile seizures in young children. AIMS Neurosci. 2022;9(4):551–558. doi: 10.3934/Neuroscience.2022032. 36660075 PMC9826744

[11] Cadet K, Boegner J, Ceneviva GD, et al. Evaluation of febrile seizure diagnoses associated with COVID-19. J Child Neurol. 2022;37(5):410–415. doi: 10.1177/08830738221086863. 35286175 PMC9086105

[12] Bentes AA, Dos Santos Junior WR, Pessoa NL, et al. Neuro-COVID-19 with or without the multisystem inflammatory syndrome (MIS-C): a single-center study. J Mol Neurosci. 2023;73(4–5):250–258. doi: 10.1007/s12031-023-02109-y. 36976476 PMC10044054

[13] LaRovere KL, Riggs BJ, Poussaint TY, et al. Neurologic involvement in children and adolescents hospitalized in the United States for COVID-19 or multisystem inflammatory syndrome. JAMA Neurol. 2021;78(5):536–547. doi: 10.1001/jamaneurol.2021.0504. 33666649 PMC7936352

[14] Wang WY, Wang YJ, An CX, et al. Multisystem inflammatory syndrome (MIS-C) with SARS-CoV-2 Omicron variant BA.2.38 in a four-year-old Chinese girl: a case report. Front Public Health. 2022;10:1021200. doi: 10.3389/fpubh.2022.1021200. 36438223 PMC9682626

[15] Wang XS, Chang HL, Tian H, et al. Epidemiological and clinical features of SARS-CoV-2 infection in children during the outbreak of Omicron variant in Shanghai, March 7–31, 2022. Influenza Other Respir Viruses. 2022;16(6):1059–1065. doi: 10.1111/irv.13044. 36043446 PMC9530495

[16] Ghahramani S, Tabrizi R, Lankarani KB, et al. Laboratory features of severe vs. non-severe COVID-19 patients in Asian populations: a systematic review and meta-analysis. Eur J Med Res. 2020;25(1):30. doi: 10.1186/s40001-020-00432-3. 32746929 PMC7396942

[17] Ou MC, Zhu JY, Ji P, et al. Risk factors of severe cases with COVID-19: a meta-analysis. Epidemiol Infect. 2020;148:e175. doi: 10.1017/S095026882000179X. 32782035 PMC7438625

[18] Mehta AA, Haridas N, Belgundi P, et al. A systematic review of clinical and laboratory parameters associated with increased severity among COVID-19 patients. Diabetes Metab Syndr. 2021;15(2):535–541. doi: 10.1016/j.dsx.2021.02.020. 33711574 PMC7896120

[19] Goldberg EE, Lin Q, Romero-Severson EO, et al. Swift and extensive Omicron outbreak in China after sudden exit from ‘zero-COVID’ policy. Nat Commun. 2023;14(1):3888. doi: 10.1038/s41467-023-39638-4. 37393346 PMC10314942

[20] You J, Tian J, Wu H, et al. Effect of tixagevimab/cilgavimab for pre-exposure prophylaxis during the China Omicron outbreak. Expert Rev Anti Infect Ther. 2023;21(12):1365–1371. doi: 10.1080/14787210.2023.2272866. 37855094

